# Intentional Replantation - Small procedure, big benefits: A report of five cases

**DOI:** 10.12669/pjms.42.(11AASC).15676

**Published:** 2026-04

**Authors:** Bibi Fatima, Tahoora Yousuf, Farhan Raza Khan

**Affiliations:** 1 Bibi Fatima,Resident Operative Dentistry & Endodontics, Department of Dentistry and Oral Health Sciences, The Aga Khan University Hospital, Karachi, Pakistan; 2 Tahoora Yousuf,Resident Operative Dentistry & Endodontics, Department of Dentistry and Oral Health Sciences, The Aga Khan University Hospital, Karachi, Pakistan; 3 Farhan Raza Khan,Professor, Department of Dentistry and Oral Health Sciences, The Aga Khan University Hospital, Karachi, Pakistan

**Keywords:** Apical periodontitis, Endodontic retreatment, Intentional replantation

## Abstract

Intentional replantation (IR) is a conservative endodontic procedure that may offer predictable results when conventional non-surgical endodontic treatment (NSET) or retreatment fails. The aim of the present report was to describe the management and outcomes of five cases of persistent apical periodontitis treated successfully with IR after failed NSET or retreatment. All the cases underwent IR involving atraumatic extraction, 3 mm root-end resection, retrograde cavity preparation, and sealing with intermediate restorative material (IRM), followed by replantation and stabilization with flexible splints or sutures. Extraoral working time was maintained under 10 minutes to preserve periodontal ligament vitality.

Follow-up periods ranged from six months to two years. All replanted teeth remained functional, asymptomatic, and radiographically stable, showing progressive or complete resolution of periapical radiolucencies. No adverse events such as ankylosis, replacement resorption, or persistent infection were observed. The favourable outcomes are attributed to careful case selection, minimal extraoral manipulation, and the use of IRM as a reliable retrograde filling material.

This series emphasizes intentional replantation as a minimally invasive, cost-effective, and biologically sound alternative to extraction or endodontic microsurgery for the preservation of natural dentition in challenging cases.

## INTRODUCTION

The primary aim of non-surgical endodontic treatment (NSET) involves the prevention or resolution of conditions affecting the pulp and peri-radicular tissues, ultimately restoring the health of the tooth along with its surrounding tissues. NSET has long-term survival and success rates, allowing individuals to retain their natural teeth. Despite these favorable outcomes, complete healing is not guaranteed, and cases of persistent apical periodontitis may occur.^1^ When initial NSET fails, alternative treatment options include non-surgical endodontic retreatment (NSERT), endodontic surgery, intentional replantation, auto-transplantation, extraction followed by prosthodontic rehabilitation.^2^

IR involves a deliberate atraumatic extraction of a tooth with history of failed primary NSET or NSERT, followed by performing sequential root resection, preparation, and sealing it hermetically with a biocompatible root-end filling material before reinserting it into the alveolus.^3^ The aim of IR is to maintain the natural tooth in function.^4^

Historically considered a last resort, IR has often been reserved for cases where other treatment modalities have already failed or are considered non-feasible. Nevertheless, recent research indicates more consistent success rates ranging from 88% to 95%, suggesting that IR is a predictable and clinically favourable option in carefully selected cases.^5^,^6^ Given these recent findings, IR could be increasingly acknowledged as a viable treatment approach.^5^

**Fig.1:**
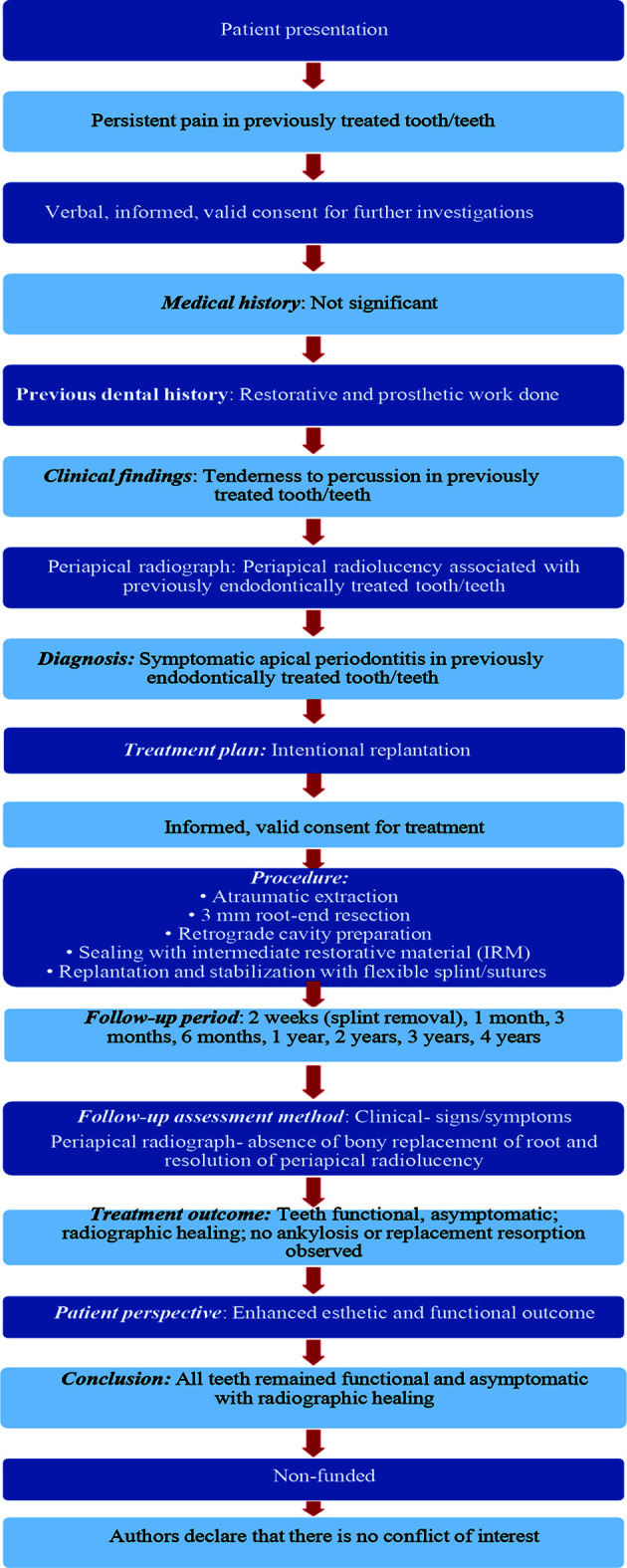
PRICE 2020 flow chart.

The present case series highlights the successful management of symptomatic apical periodontitis of teeth treated through IR, leading to the restoration of dental function and aesthetics for the patients. This case series was prepared in accordance with the PRICE (Preferred Reporting Items for Case reports in Endodontics) 2020 guidelines to ensure comprehensive and transparent reporting (Fig.1).^7^

### Case-1:

**Figure 2:**
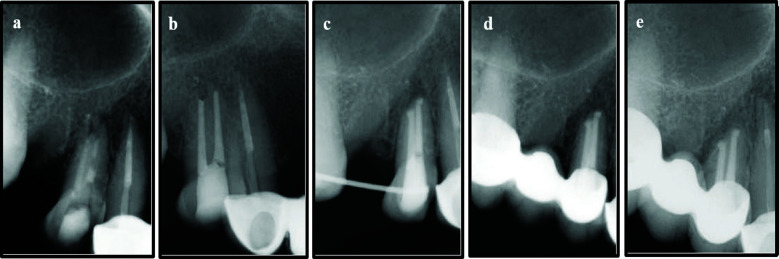
a) Primary non-surgical endodontic treatment 14; b) Non-surgical endodontic retreatment; c) Immediate post intentional replantation of tooth 14; d) Six-month follow-up; e) One year follow-up.

A 62 years old healthy female presented to our clinics with pain and swelling on the right maxilla. Extra-orally, moderate facial swelling with normal mouth opening was noted. Intra-orally, she exhibited poor oral hygiene, unsatisfactory prostheses in both arches, multiple carious teeth, a faulty restoration on tooth 14, and a missing tooth 15. Radiographs showed inadequately treated root canals in tooth 14 (Fig.2a). A diagnosis of symptomatic apical periodontitis in a previously treated tooth 14 was established.

NSERT was initiated under local anaesthesia (2% lidocaine with 1:100,000 epinephrine) and rubber dam isolation. After removal of previous filling material, instrumentation was completed to working length with ProTaper Next (PTN) X3 (30/07). The tooth was dried, temporized, and analgesics prescribed. At the second visit (two days post-retreatment), a flare-up was managed by removing the temporary restoration, cleaning the canals, and re-temporizing. At the third visit (after one week), the patient was asymptomatic, and obturation was completed using the single- cone technique followed by composite restoration (Fig.2b).

Six months later, the patient reported with severe pain on palpation and biting. Despite adequate obturation, persistent symptomatic apical periodontitis was diagnosed. Treatment options, including endodontic microsurgery, IR, and extraction with delayed implant, were discussed. The patient opted for IR, and a written informed consent was obtained.

**Figure 3:**
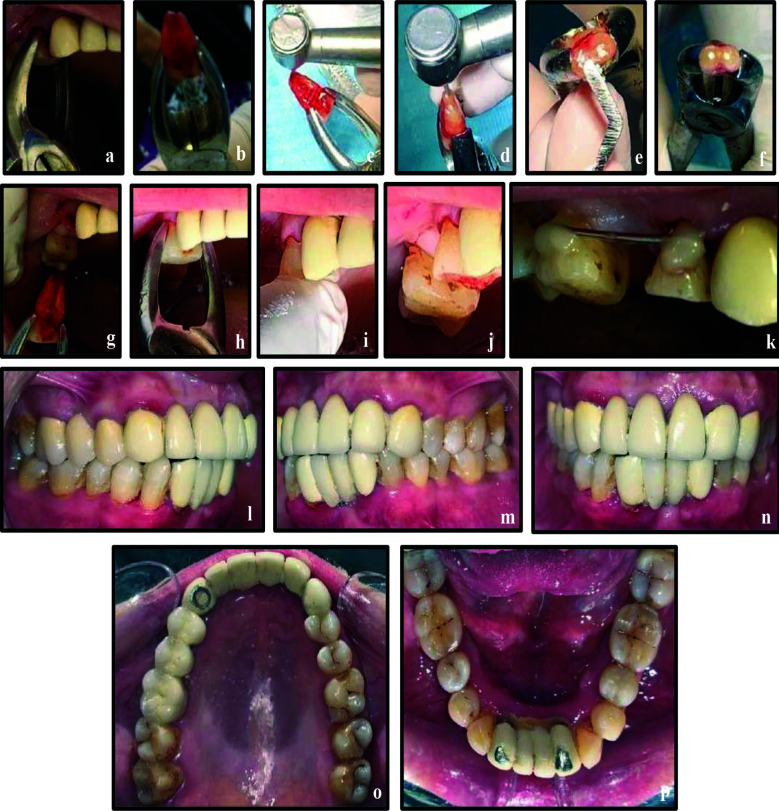
a & b, Atraumatic extraction of tooth 14; c, Root-end resection; d, Root-end preparation; e & f, Retrograde filling; g, h, i & j, Tooth placement in its socket; k, Splinting of tooth 14 to tooth 16 with flexible splint; l, m, n, o, Six months post intentional replantation with tooth 14 serving as an abutment for fixed dental bridge 14-16; p, Mandibular occlusal view.

Under local anaesthesia, tooth 14 was atraumatically extracted via dental forceps, 3 mm of root end resected using tapered fissure bur (Mani Inc., Japan) with a high-speed handpiece under water cooling. Retrograde cavity was prepared, and intermediate restorative material (IRM) was backfilled into the prepared root end cavity. The extra-oral procedure was completed within seven minutes, and the tooth was replanted into the prepared socket. Primary stability was achieved by splinting to tooth 16 with a flexible wire (stainless steel- 0.4mm) (Fig.2c, Fig.3a-k), and the patient advised chlorhexidine rinses and a soft diet for one-two weeks.

At the two week follow-up, the patient presented with tooth 14 being asymptomatic and exhibiting Grade-I mobility. Since the splinting was supported only on one distant tooth due to the absence of adjacent healthy tooth, the splinting period was extended to additional two weeks and was removed at the fourth week.

At the six months follow-up, the tooth exhibited normal mobility, no tenderness to percussion or palpation, and radiographs showed no evidence of root resorption. Periodontal probing depths remained within normal limits. At this visit, the patient expressed interest in replacing missing tooth 15. Various treatment options were discussed, and the patient opted for a fixed dental bridge. Teeth 14 and 16 were prepared as abutments, impressions were taken, and a provisional restoration was placed. One week later, a porcelain-fused-to-metal (PFM) bridge spanning teeth 14–16 was delivered (Fig.2d, Fig.3l–p). At the one-year recall, bone healing was evident at the apex of tooth 14 (Fig.2e), which remained asymptomatic, while the prosthesis demonstrated satisfactory function and aesthetics.

### Case-2:

**Figure 4:**
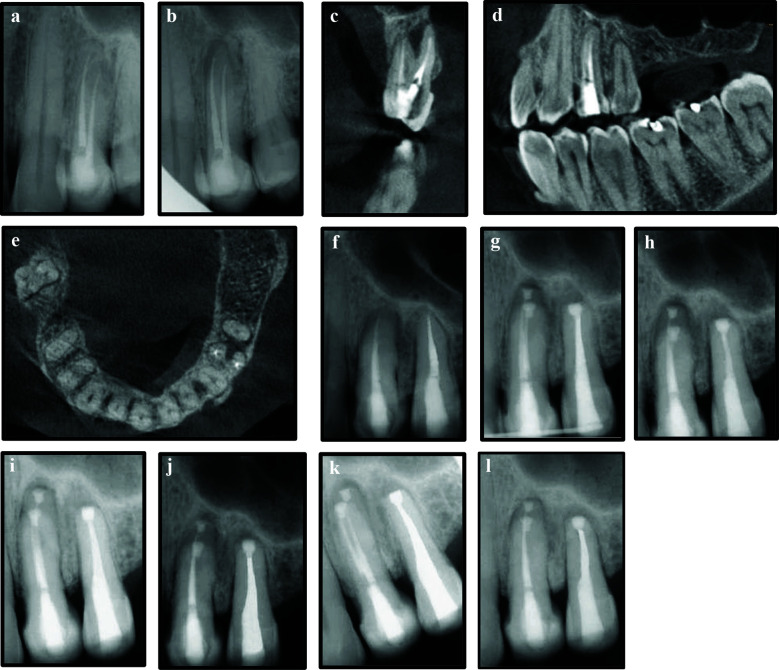
a, Periapical radiograph immediate post non-surgical endodontic treatment of tooth 24; b, Four years post-treatment; c, d & e, Cone beam computed tomographic cross-sectional, sagittal and axial views at the four year follow-up visit; f, Periapical radiograph immediate post non-surgical endodontic retreatment of tooth 24 and primary endodontic treatment of tooth 25; g, Intentional replantation of teeth 24 and 25; h, One month follow-up; i, Three months follow-up; j, Six months follow-up; k & l, 12 months and 16 months follow-up respectively.

A 66 years old healthy male presented with pain on biting in the left maxillary region. He had a history of NSET in tooth 24 (Fig.4a) performed four years ago. Clinical examination revealed multiple missing maxillary teeth, for which the patient was wearing a cast partial denture. Tooth 24 was crowned, and both teeth 24 and 25 were tender to percussion. Periapical radiograph showed a periapical radiolucency associated with tooth 24 (Fig.4b), while pulp sensibility testing of tooth 25 yielded a negative response. Based on these findings, a diagnosis of symptomatic apical periodontitis in a previously treated tooth 24 and necrotic pulp with symptomatic apical periodontitis in tooth 25 was established. Cone-beam computed tomography (CBCT) was taken to confirm the diagnosis (Fig.4c, d, e).

NSERT of tooth 24 was carried out through the existing crown, and primary endodontic treatment of tooth 25 was performed under local anaesthesia and rubber dam isolation (Fig.4f). The patient presented seven months later with persistent symptoms in both the teeth. After discussing alternative treatment options as in case 1, IR was planned for both the teeth, and informed consent was obtained from the patient.

IR was performed following the same protocol as for case 1. The total extra-oral time was 10 minutes, and the teeth were splinted to adjacent teeth 21, 22 and 23 using a flexible wire (stainless steel- 0.4mm) [Fig.4g]. The patient was instructed to maintain a soft diet, use 0.12% chlorhexidine rinses, and avoid wearing his cast partial denture for four weeks.

**Figure 5:**
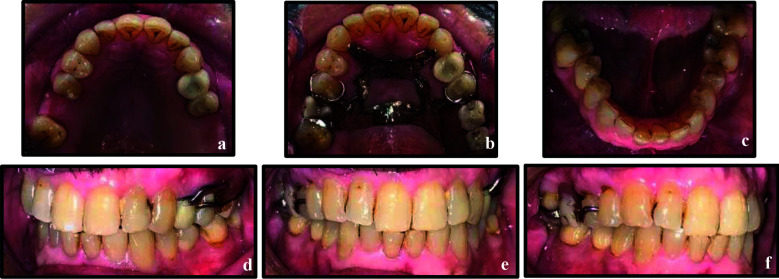
a & b, Maxillary occlusal views post intentional replantation of teeth 24 and 25 and supporting cast partial denture respectively; c, Mandibular occlusal view; d, e & f, left buccal, frontal and right buccal views respectively.

The splint was removed after three weeks, and the teeth exhibited grade I mobility with slight tenderness to percussion. By the one and three-month follow-up visits (Fig.4h, i), the teeth demonstrated normal mobility and no tenderness to percussion; however, radiographic healing of the periapical lesion remained incomplete. The patient was advised to resume wearing his maxillary cast partial denture. At the six and 12 months, radiographs showed favorable bone healing, and by 16 months, the periapical lesion exhibited almost complete resolution (Fig.4j- l, Fig.5a-f). The patient continues to remain under regular clinical and radiographic follow-up.

### Case-3:

**Figure 6:**
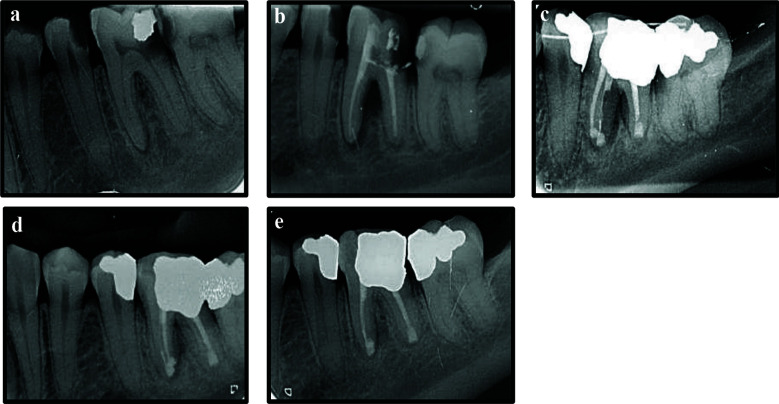
a, Preoperative periapical radiograph showing teeth 34 to 37; b, Non-surgical endodontic treatment in tooth 36 with separated instrument in mesiolingual canal extruding beyond the apex; c, Immediate post intentional replantation and splinting of tooth 36 to adjacent teeth; d, Six-months follow-up; e, Two years follow-up.

A 24 years old healthy female presented with severe pain in the left mandibular region. Clinical examination revealed carious lesions in teeth 35 and 37, a recurrent decay and tenderness to percussion associated with tooth 36. Pulp sensibility testing showed positive, non-lingering responses in teeth 35 and 37, while tooth 36 was non-responsive. Radiographic evaluation demonstrated a periapical radiolucency associated with tooth 36 (Fig.6a). Based on these findings, a diagnosis of pulp necrosis with symptomatic apical periodontitis was established. After taking informed consent, NSET was initiated for tooth 36 under rubber dam isolation. The same protocol was followed as for cases 1 and 2. During instrumentation, a PTN X2 file (4 mm fragment) fractured in the mesiolingual canal and extruded beyond the apex. Due to its location, retrieval of the separated instrument was difficult; therefore, the treatment was completed using the single cone obturation technique (Fig.6b), followed by core buildup with an amalgam restoration.

The patient was informed about the instrument separation, and different management options were discussed. After informed consent, IR was carried out following the same protocol as in earlier cases. The tooth was atraumatically extracted, managed extra-orally, and replanted within 10 minutes. It was stabilized using a flexible splint attached to the adjacent teeth. Oral hygiene instructions were provided, and the patient was recalled after two weeks, during which the splint was removed. The tooth was slightly tender to percussion, with grade I mobility at the two-week follow-up period.

**Figure 7:**
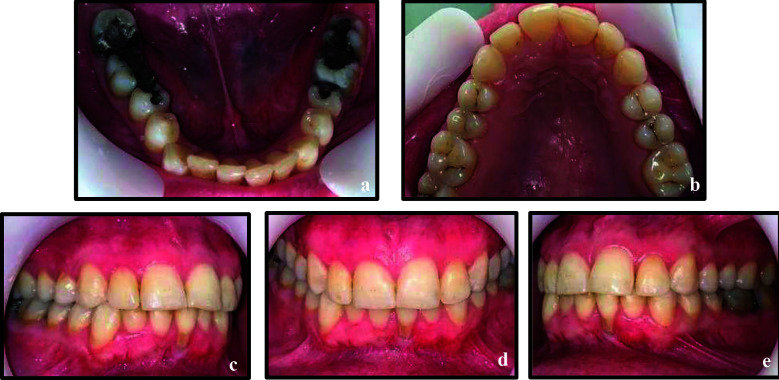
Post-operative images at two years follow-up. a, Mandibular occlusal view; b, Maxillary occlusal view; c, d, & e, Right buccal, frontal and left buccal views respectively.

The patient was advised regular clinical and radiographic follow-up; however, she reported only at six months and two years post-treatment. At the six-months visit (Fig. 6d), the tooth exhibited normal mobility and was non-tender to percussion. At the two-year follow-up, the tooth remained asymptomatic, functional, and retained in the arch, with clear clinical and radiographic signs of healing, and no evidence of root resorption or periapical pathology (Fig.6e, Fig.7a-e).

### Case-4:

**Figure 8:**
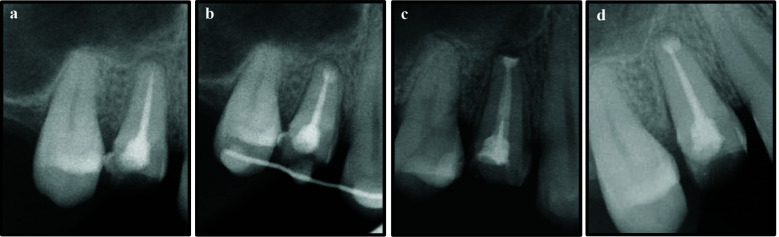
a, Preoperative radiograph showing previously treated tooth 14; b, Immediate post intentional replantation and splinting of tooth 14; c & d, Three months and six months follow-up respectively.

A 27 years old female, with no known co-morbids, presented to the dental department of our hospital with the complaint of severe pain on biting in the right upper tooth, for the past one year. Upon taking detailed history, the patient mentioned that the tooth underwent NSET twice at some other institute, but the pain persists. Clinical examination showed tooth 14 had been prepared for a full-coverage restoration, exhibited tenderness to percussion and palpation, but demonstrated normal probing depths and absence of mobility. Periapical radiograph revealed well obturated canals with associated radiolucency surrounding the apex of the same tooth (Fig.8a). The diagnosis of symptomatic apical periodontitis in previously endodontically treated tooth 14 was made and after a mutual decision of the patient and the dentist, IR was planned as a treatment option for tooth 14.

**Figure 9:**
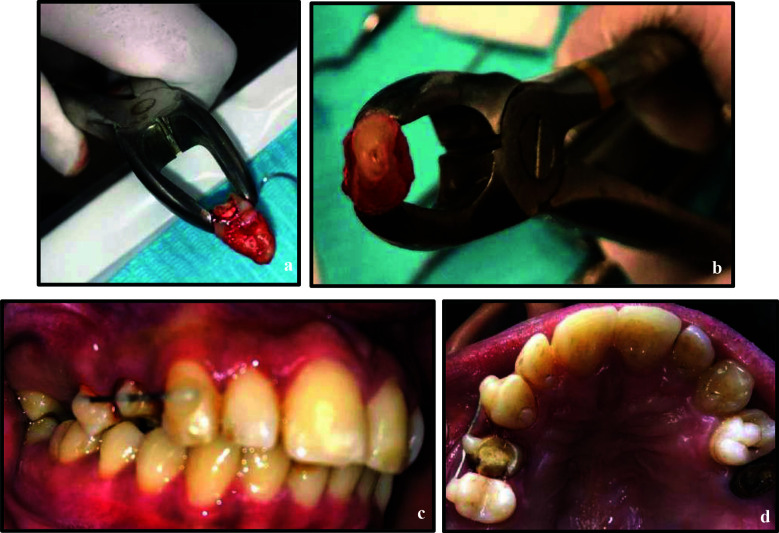
a, Atraumatic extraction of tooth 14; b, Root-end resection and retrocavity preparation; c & d, Splinting of tooth 14 to adjacent teeth.

Informed consent was obtained, and after the administration of local anesthesia, the same protocol for IR was followed as in the earlier three cases (Fig.9). The entire extra-oral procedure was completed within 10 minutes. The tooth was then replanted in the previously prepared alveolus and stabilized by splinting to adjacent teeth 13 and 15, via stainless steel wire (SS- 0.4mm) and composite resin (Fig.8b, Fig.9c, d). The patient was given oral hygiene instructions and recalled after two weeks, at which time the splint was removed. At the three-month follow-up, the tooth was stable with no mobility but exhibited slight tenderness to percussion. By the six-month review, the tooth demonstrated normal mobility, absence of symptoms, and probing depths within normal limits. A full-coverage restoration was advised for tooth 14, and the patient remains under regular follow-up.

### Case-5:

**Figure 10:**
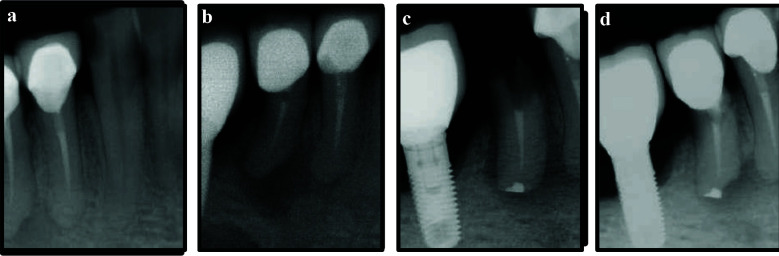
a, Periapical radiograph of tooth 45 immediate post-endodontic treatment; b, Two years post-endodontic treatment; c, Immediate post intentional replantation; d, Five months follow-up.

**Figure 11:**
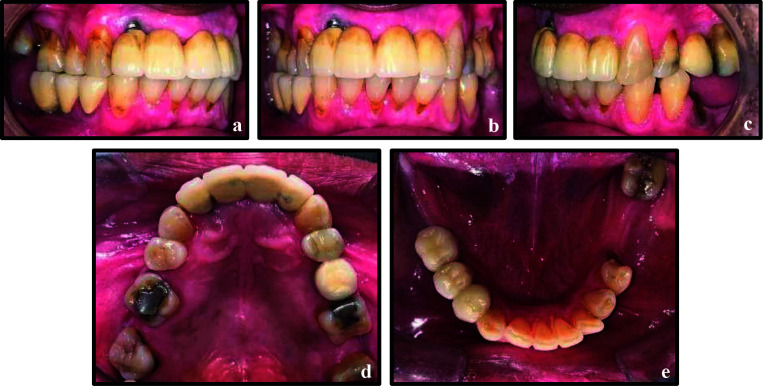
Pre-operative images: a, b, & c, right buccal, frontal and left buccal views; d, maxillary occlusal; e, mandibular occlusal view.

A 49 years old healthy male presented to the dental department of our hospital with the complaint of pain and swelling in the lower right quadrant. He had undergone NSET in tooth 45, two years ago (Fig.10a). Clinical examination showed a porcelain fused to metal (PFM) crown on tooth 45 (Fig.11a-e), with associated tenderness to percussion. Periapical radiograph showed root canal obturation short of the radiographic apex, with a periapical radiolucency (Fig.10b). The under-obturation was attributed to sclerosed canals in the apical third. The diagnosis of symptomatic apical periodontitis in previously endodontically treated tooth 45 was made. Extraction was initially advised due to the guarded restorative prognosis; however, as the patient strongly preferred to retain the tooth, IR was mutually agreed upon as the treatment of choice.

**Figure 12:**
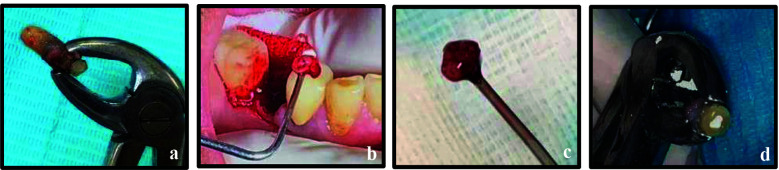
Intra-operative images: a, Atraumatic extraction of tooth 45, b; Curettage of extraction socket; c, Removal of granulation tissue; d, Retrograde filling.

After taking informed consent and local anaesthesia administration, the tooth was atraumatically extracted, during which the prosthetic crown separated from the tooth (Fig.12a). The same IR protocol was followed as in the previous cases. The socket was curetted at the apical third, and granulation tissue was removed before replantation of the tooth (Fig.12b & c). The entire extra- oral procedure was completed within 10 minutes. The tooth was replanted into the prepared alveolus (Fig.10c) and stabilized with Vicry l 3-0 sutures, after which PFM crown was recemented. The patient was provided with oral hygiene instructions and recalled after two weeks; at which time the sutures were removed. Owing to travel commitments, the patient returned for follow-up after five months, at which time the tooth was asymptomatic with normal mobility and probing depths. Periapical radiograph of tooth 45 demonstrated reduction in the size of the periapical radiolucency (Fig.10d) indicating healing of the periapical tissues. The patient remains under regular follow-up.

## DISCUSSION

Intentional replantation (IR) offers a simple, less invasive, efficient, and more cost-effective alternative to peri-radicular surgery.^8^ Favourable outcomes are typically indicated by clinical comfort, absence of symptoms, restoration of function, and radiographic resolution of periradicular lesion. Successful healing depends on several factors, such as reducing the extraoral time (<15 minutes), atraumatic tooth extraction, and adequate apical seal provided by proper depth and compaction of the material, and judicious case selection.^9^ Minimizing extraoral time and atraumatic manipulation are especially important, because these factors prevent cellular dehydration and aid in maintaining the cell vitality of periodontal ligament necessary for periradicular healing and to reduce the risk of replacement resorption, ankylosis, and internal or external root resorption.^10^ Since traumatically extracted teeth are unsuitable candidates for IR, meticulous case selection is crucial to ensure that extraction and reinsertion can be performed smoothly, without damaging the buccal or lingual plates of the alveolar bone.^9^

In the current cases, IR was considered a suitable treatment option for multiple reasons, including operator confidence, limited intraoral access, coronal restrictions, failed but adequately performed orthograde NSET, and difficulty accessing the site for periapical surgery. Patient-related factors including financial limitations, fear of surgery, preference for tooth retention, and clinician recommendation, also influenced treatment choice. In the present cases, despite a short follow-up period except for case-3, all the replanted teeth showed favorable healing with primary stability, absence of mobility, and no signs of infection. These outcomes can be attributed to atraumatic handling, reduced extraoral time, and appropriate case selection, making IR a cost-effective, minimally invasive, and biologically favorable option for preserving the natural teeth.

Unlike most previous studies on IR, the present cases employed IRM, a zinc oxide eugenol-based cement that has been widely used as a root-end filling material for decades. The choice of root- end filling material is critical, as properties such as sealing ability, antibacterial activity, biocompatibility, and potential to promote hard tissue repair directly influence treatment outcomes.^11^ IRM demonstrates reliable sealing, antibacterial effects due to its eugenol content, ease of manipulation, availability, and lower cost compared with newer biomaterials, factors that make it a practical and easily accessible in many clinical settings.^12^,^13^ Although calcium silicate- based cements such as Mineral Trioxide Aggregate (MTA) are widely regarded as the gold standard in contemporary practice due to their excellent sealing ability, bioactivity, biocompatibility, and proven capacity to promote cementogenesis and periradicular healing,^14^ a systematic review by Abou ElReash et al. reported that there is no significant difference in success rates between MTA and IRM, with neither material demonstrating clear superiority as a retrograde filling material.^15^ Therefore, IRM continues to be a cost-effective and clinically reliable alternative, particularly in situations and environments where economic or logistical constraints limit the use of advanced biomaterials. This is particularly relevant in resource restraint countries especially the ones in low- middle income bracket.

In our series, IR combined with extraoral endodontic root-end surgery using IRM cement achieved predictable clinical and radiographic success within a limited follow-up period. This result is somewhat higher than that reported by Koenig et al. (82%),^16^ and is comparable to the 89% reported by Peer et al.^17^ and >90% success documented by Cho et al.^18^ Collectively, these findings suggest that when performed under optimal conditions and with careful case selection, IR can achieve outcomes on par with or superior to those reported in the literature, even in the short term. Another clinical consideration relates to preparation of the alveolar socket. While curettage of the socket wall remains controversial, as it may remove residual periodontal ligament fibers critical for healing, in the present report, curettage was limited to the apical region to eliminate granulation or cystic tissue while preserving the remaining socket walls. This conservative approach may have further contributed to the favourable healing outcomes observed. Extraoral working time also played an important role: in our cases, the extraoral phase was limited to 7–10 minutes. Since extended extraoral time is strongly associated with IR failures due to resorption or ankylosis,^10^ adhering to this short timeframe likely contributed to success. Our findings are consistent with the study by Abou ElReash et al.^15^ and Bakhsh et al.^19^ in which the extra-oral time was between 8–14 minutes and 10 minutes respectively, and further align with evidence showing that IR survival decreases by approximately 1.7-fold when extraoral time exceeds 15 minutes.^20^

This study is not without limitations. The short to medium term follow-up restrict the ability to evaluate the long-term prognosis of IR. Furthermore, the use of IRM instead of calcium silicate– based cements may be considered a limitation, given the superior biological properties of MTA and Biodentine. Root-end resection was also performed using a tapered fissured bur rather than ultrasonic instruments, which could have affected the precision of preparation and sealing.

Nevertheless, the present findings demonstrate promising short-term outcomes. Previous studies have reported comparable survival rates for IR when evaluated against endodontic microsurgery and non-surgical retreatment, suggesting that IR may serve as a reliable alternative in carefully selected cases.^6^,^21^ Moreover, as implants and other prosthetic options continue to rise in popularity, IR presents a tooth-preserving, biologically favorable, and cost-effective treatment modality that avoids some of the complications associated with prosthetic rehabilitation.^20^ However, the absence of standardized clinical protocols, variability in operator skill, and the need for careful patient selection currently limit its widespread acceptance. Future research should focus on well-designed clinical trials with larger samples, extended follow-up, and direct comparisons of biomaterials such as IRM, MTA, and Biodentine, to develop evidence-based guidelines and further establish intentional replantation as a predictable treatment modality.

## CONCLUSION

The present report of five cases demonstrates that intentional replantation can provide predictable and favorable outcomes in the management of persistent apical periodontitis following failed NSET. When performed with minimal extraoral time, careful handling, and appropriate case selection, IR serves as a viable, tooth-preserving alternative to endodontic surgery or extraction, especially in patients seeking cost-effective and biologically conservative care. While the short follow-up in some cases limits long-term prognostic insights, the results highlight IR as a practical treatment modality that deserves greater consideration in contemporary endodontic practice.

### Author`s Contributions:

**BF:** Conception and design of the study, clinical management of cases, drafting of the manuscript. **TY:** Data acquisition and interpretation, literature review, critical revision of the manuscript.

**FRK:** Supervision, interpretation of findings, critical review, and final approval of the manuscript.

All authors have read and approved the final version and agree to be accountable for all aspects of the work.

## Availability of data and materials:

Data sharing not applicable to this article as no datasets were generated or analyzed during the current study.
